# Perirhinal cortex and temporal lobe epilepsy

**DOI:** 10.3389/fncel.2013.00130

**Published:** 2013-08-29

**Authors:** Giuseppe Biagini, Margherita D'Antuono, Ruba Benini, Philip de Guzman, Daniela Longo, Massimo Avoli

**Affiliations:** ^1^Laboratory of Experimental Epileptology, Department of Biomedical, Metabolic and Neural Sciences, University of Modena and Reggio EmiliaModena, Italy; ^2^Montreal Neurological Institute and Departments of Neurology and Neurosurgery and of Physiology, McGill UniversityMontréal, QC, Canada; ^3^Faculty of Medicine and Dentistry, Department of Experimental Medicine, Sapienza University of RomeRoma, Italy

**Keywords:** cholecystokinin, hippocampal formation, interneurons, neuropeptide Y, parvalbumin, perirhinal cortex, pilocarpine, temporal lobe epilepsy

## Abstract

The perirhinal cortex—which is interconnected with several limbic structures and is intimately involved in learning and memory—plays major roles in pathological processes such as the kindling phenomenon of epileptogenesis and the spread of limbic seizures. Both features may be relevant to the pathophysiology of mesial temporal lobe epilepsy that represents the most refractory adult form of epilepsy with up to 30% of patients not achieving adequate seizure control. Compared to other limbic structures such as the hippocampus or the entorhinal cortex, the perirhinal area remains understudied and, in particular, detailed information on its dysfunctional characteristics remains scarce; this lack of information may be due to the fact that the perirhinal cortex is not grossly damaged in mesial temporal lobe epilepsy and in models mimicking this epileptic disorder. However, we have recently identified in pilocarpine-treated epileptic rats the presence of selective losses of interneuron subtypes along with increased synaptic excitability. In this review we: (i) highlight the fundamental electrophysiological properties of perirhinal cortex neurons; (ii) briefly stress the mechanisms underlying epileptiform synchronization in perirhinal cortex networks following epileptogenic pharmacological manipulations; and (iii) focus on the changes in neuronal excitability and cytoarchitecture of the perirhinal cortex occurring in the pilocarpine model of mesial temporal lobe epilepsy. Overall, these data indicate that perirhinal cortex networks are hyperexcitable in an animal model of temporal lobe epilepsy, and that this condition is associated with a selective cellular damage that is characterized by an age-dependent sensitivity of interneurons to precipitating injuries, such as *status epilepticus*.

## Background

**Figure 1:**
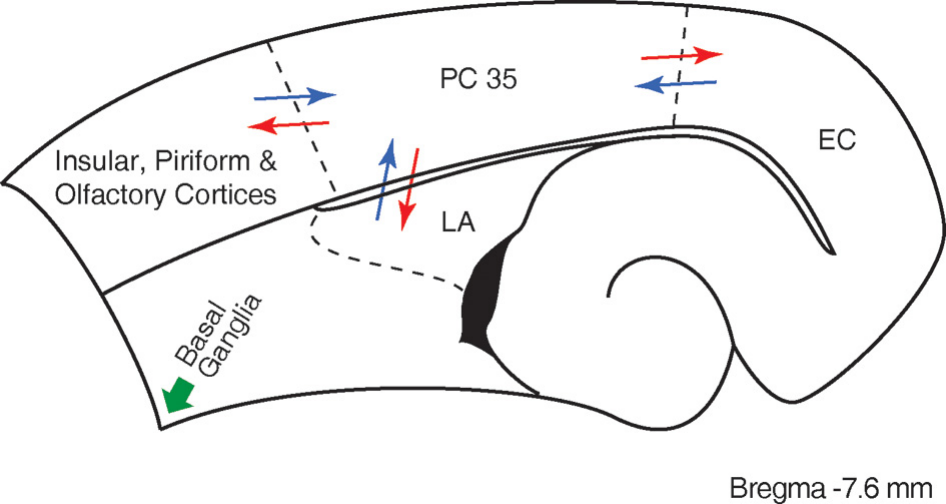
**Scheme of the main afferent/efferent connections of the perirhinal cortex (PC, area 35) under physiological conditions**. The drawing corresponds to a section taken at 7.6 mm from the bregma according to the Paxinos and Watson (2007) atlas. Major afferents projections to the perirhinal cortex (blue arrows) originate from olfactory insular and piriform cortices, lateral amygdala (LA) and entorhinal cortex (EC); conspicuous efferent projections of the perirhinal cortex (red arrows) are directed to these areas as well. Note that subcortical efferents from the perirhinal cortex (green arrows) terminate in several brain regions, including the basal ganglia (cf., Furtak et al., 2007).

The perirhinal cortex is a limbic structure that is closely interconnected with the lateral entorhinal cortex, the amygdala, and with unimodal and polymodal association cortices (Suzuki and Amaral, 1994; Burwell et al., 1995; Kealy and Commins, 2011). Hippocampal networks exchange information with the neocortex through the rhinal cortices (Van Hoesen, 1982; Naber et al., 1999; Kealy and Commins, 2011) (Figure 1), and it has been consistently demonstrated that the perirhinal cortex is intimately involved in learning and memory (Zola-Morgan et al., 1989; 1993; Murray et al., 1993; Suzuki et al., 1993; Suzuki, 1996; Weintrob et al., 2007; Kealy and Commins, 2011).

Knowledge on the memory functions of the perirhinal cortex has been obtained from patients presenting with temporal lobe epilepsy. Initial observations in patients undergoing epilepsy neurosurgery reported vivid recollection or sensation of familiarity known as *déjà vu* and *déjà vécu* when the temporal lobe was electrically stimulated (Penfield and Perrot, 1963; Bancaud et al., 1994). In addition, Bartolomei et al. (2004) found that similar experiential phenomena were elicited more frequently by stimulating the rhinal cortices than the amygdala or the hippocampus. Specifically, they reported that *déjà vu* was obtained following stimulation of the entorhinal cortex, whereas reminiscence of memories occurred during perirhinal cortex stimulation.

The perirhinal cortex has also been investigated for the potential contribution of this region to ictogenesis in the limbic system (McIntyre and Plant, 1989; Kelly and McIntyre, 1996). Pioneering investigations based on the kindling protocol identified the amygdala and the piriform cortex as major epileptogenic areas (Kelly and McIntyre, 1996). For this reason, McIntyre and his collaborators proposed an *in vitro* amygdala-piriform slice preparation to characterize the properties of these limbic areas. Because of the limited spontaneous epileptiform activity observed in the slice preparation, they challenged neuronal networks with a modified bathing medium, devoid of magnesium; this experimental procedure revealed a prominent epileptiform activity that was generated in the perirhinal cortex (McIntyre and Plant, 1989). These findings gave rise to a series of *in vivo* experiments demonstrating that: (i) the piriform cortex is not crucial in the spread of seizures originated in the hippocampus; (ii) the perirhinal cortex is kindled in a faster manner compared to other limbic regions and, above all, presents with the lowest latency to seizure spread to frontal cortex motor areas; and (iii) the posterior region of the perirhinal cortex is critical to the propagation of hippocampal seizures (Kelly and McIntyre, 1996).

Compared to other limbic areas, the perirhinal cortex remains overlooked, and in particular detailed information on its dysfunctional characteristics are scarce. Over the last decade, however, some studies have begun to unveil the fundamental electrophysiological properties and the morphological features of perirhinal cortex cells (Bilkey and Heinemann, 1999; Faulkner and Brown, 1999; Beggs et al., 2000; D'Antuono et al., 2001; Furtak et al., 2007). In addition, new pathophysiological roles for this limbic structure in epileptogenesis and ictogenesis are emerging. Our paper is aimed at: (i) reviewing the electrophysiological characteristics of neurons that are recorded in the perirhinal cortex in an *in vitro* slice preparation; (ii) summarizing data regarding the ability of perirhinal cortex neuronal networks to generate epileptiform discharges when challenged with acute epileptogenic pharmacological procedures; (iii) highlighting the changes in neuronal excitability that occur in the pilocarpine model of temporal lobe epilepsy; and (iv) elucidating the contribution of selective interneuron subtype damage in promoting epileptogenesis.

## Fundamental intrinsic and synaptic properties

**Figure 2:**
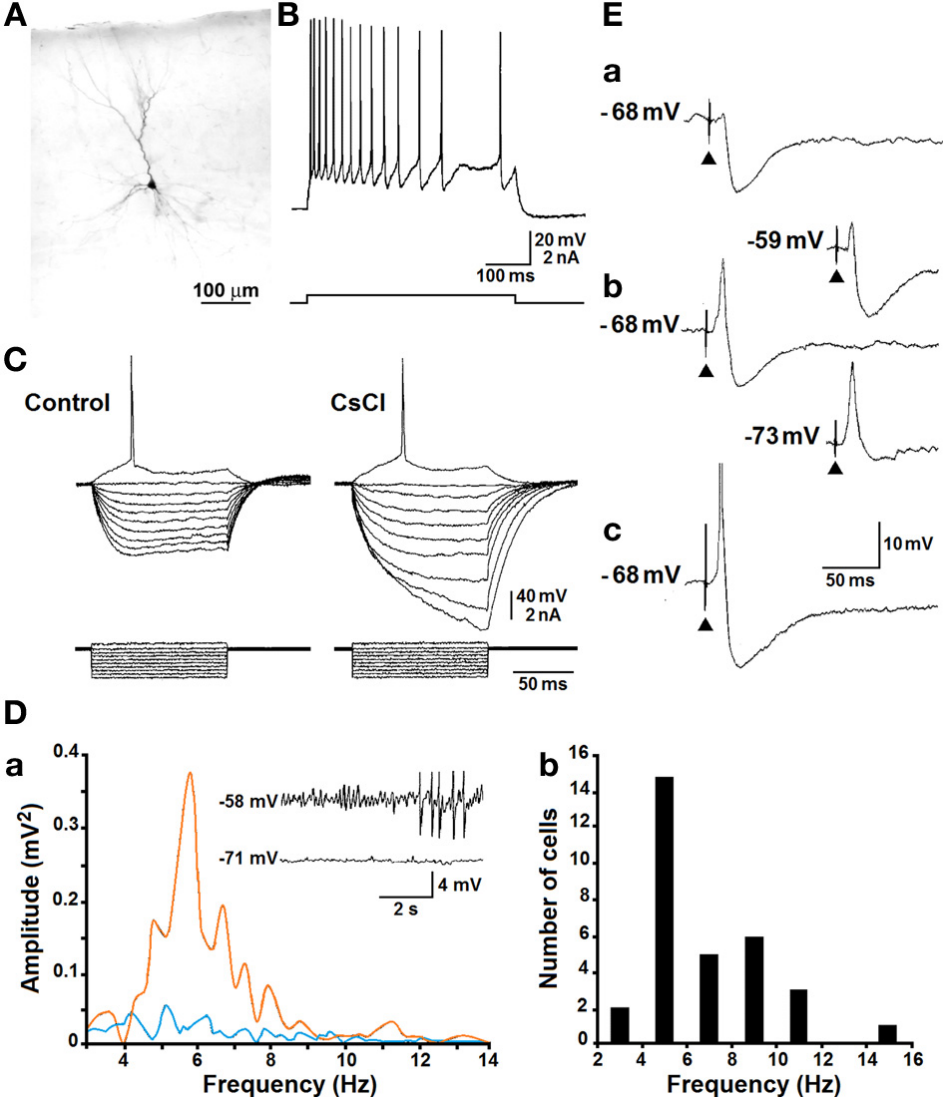
**(A)** Photomicrograph of a neurobiotin-filled perirhinal cortex cell that corresponds to an upright pyramidal neuron (cf., Furtak et al., 2007). Scale bar, 100 μm. **(B)** Regular repetitive firing with adaptation is generated by a pyramidal-like neuron recorded in the perirhinal cortex during injection of a pulse of depolarizing current. **(C)** Effects induced by bath application of CsCl (3 mM) on the voltage responses to intracellular current pulses; note that under control conditions stepwise hyperpolarization of the membrane leads to the appearance of a sag toward the resting level as well as that addition of CsCl to the bath increases the neuronal input resistance and abolishes the sag. Bottom traces represent current monitor. **(D)** Power spectra of the intracellular signals recorded in the cell shown in the insets at two different levels of depolarization are illustrated in **(a)**; note that the spectrum obtained from the signal recorded at −58 mV (orange line) is characterized by a peak at ~5.5 Hz. In **(b)**, histogram of the peak frequencies of membrane oscillations recorded in 29 perirhinal cortical neurons. **(E)** Responses recorded intracellularly from a perirhinal cortex neuron following local single-shock extracellular stimuli of progressively increasing strength (from **a** to **c**). Inserts in panel **(b)** illustrate the intracellular responses recorded during injection of depolarizing (−59 mV trace) or hyperpolarizing (−73 mV trace) current.

Intracellular studies performed in the perirhinal cortex have shown that neurons include fast-spiking, burst-spiking and regular-spiking cells (Kelly and McIntyre, 1996; Faulkner and Brown, 1999; Kealy and Commins, 2011). In addition, Beggs et al. (2000) have described late-spiking pyramidal cells that are capable of generating delayed action potential discharges, and proposed that these neurons may play a role in encoding “long-time intervals” during associative learning. By employing sharp intracellular recordings (D'Antuono et al., 2001; Benini et al., 2011), we found that most of the neurons recorded in the perirhinal cortex correspond morphologically to spiny pyramidal cells and are regularly firing (Figures 2A,B). These neurons generate several types of sub-threshold responses during injection of intracellular current pulses including: (i) tetrodotoxin-sensitive inward rectification in the depolarizing direction (not illustrated) and (ii) Cs^+^-sensitive inward rectification during injection of hyperpolarizing current pulses (Figure 2C). In addition, the repetitive firing generated by these neurons is characterized by adaptation and is followed by a slow after-hyperpolarization upon termination of the depolarizing current pulse (Figure 2B). We have also found that both phenomena are greatly reduced by application of Ca^2+^ channel blockers, indicating that Ca^2+^-activated K^+^ conductances play an important role in controlling the intrinsic excitability of pyramidal cells in the perirhinal cortex. These intrinsic properties are indeed similar to those demonstrated in principal cells recorded intracellularly in several cortical structures (Constanti and Galvan, 1983; Stafstrom et al., 1985; Spain et al., 1987; Mattia et al., 1997).

Pyramidal neurons in the perirhinal cortex are also capable of generating voltage-gated, subthreshold membrane oscillations at 5–12 Hz during steady injection of depolarizing current (Bilkey and Heinemann, 1999). As illustrated in Figure 2Da (inserts), when neurons were recorded at resting membrane potential (more negative than −70 mV), no significant oscillatory activity was observed; however, when they were depolarized with injection of steady intracellular current, sinusoidal-like oscillations became evident along with “clustered” or “tonic” action potential firing. This phenomenon is further identifiable in the power spectrum of the intracellular signals recorded at −70 and −58 mV (Figure 2Da), while the plot histogram in Figure 2Db summarizes the peak frequencies of the subthreshold membrane oscillations recorded from several perirhinal cortical cells. It should be emphasized that as reported in entorhinal cortex or subicular cells (Alonso and Llinas, 1989; Mattia et al., 1997), this voltage-dependent oscillatory activity persisted during blockade of glutamatergic and γ-aminobutyric acid (GABA)ergic transmission with specific receptor antagonists as well as during application of Ca^2+^ channel blockers. However, it disappeared during application of tetrodotoxin suggesting that voltage-gated Na^+^ electrogenesis contributes to this oscillatory phenomenon.

As shown in Figure 2E, perirhinal principal cells generate synaptic potentials with polarity and amplitude that depend on the intensity of the extracellular stimulus; thus, stimuli at threshold strength (Figure 2Ea) often induced a hyperpolarizing inhibitory postsynaptic potential (IPSP) while, at progressively higher intensities, an excitatory postsynaptic potential (EPSP)-IPSP sequence (Figure 2Eb) and eventually an EPSP-single action potential (Figure 2Ec) occurred. Moreover, these responses changed in amplitude during injection of depolarizing or hyperpolarizing current (Figure 2Eb) and the early hyperpolarizing component of the IPSP was characterized by reversal potential values at approximately −80 mV (not illustrated).

Overall these findings indicate that the intrinsic properties of principal cells in the perirhinal cortex reproduce those reported for cortical pyramidal cells in several areas of the brain. The presence of fast-spiking cells (Faulkner and Brown, 1999) that are known to release GABA is mirrored by the ability of principal neurons in the perirhinal cortex to generate robust inhibitory responses both spontaneously and following electrical stimuli (Benini et al., 2011).

## Epileptiform synchronization *in vitro*

**Figure 3:**
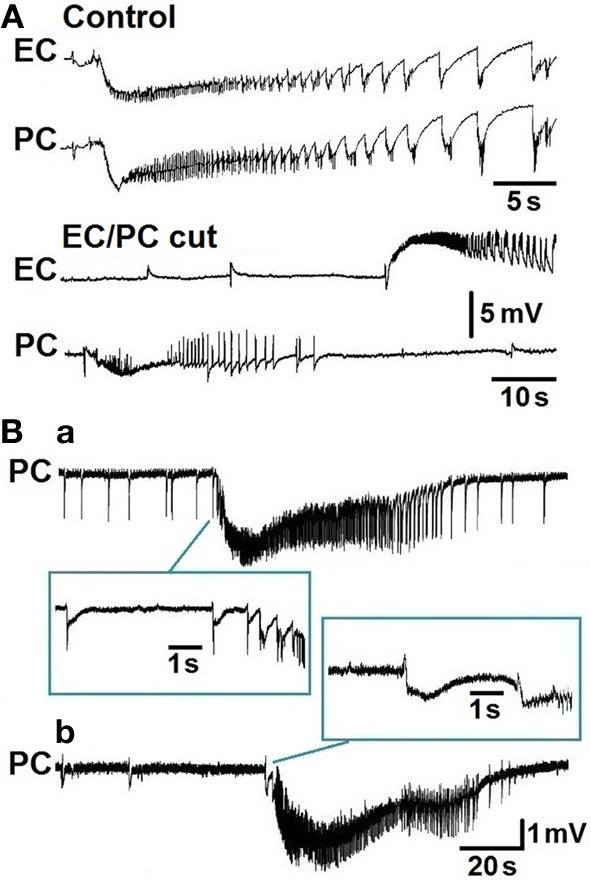
**(A)** Ictal discharges occur synchronously in entorhinal (EC) and perirhinal (PC) cortices in an *in vitro* brain slice treated with 4-aminopyridine (Control panel). Note that cutting the connections between entorhinal and perirhinal areas causes the occurrence of independent ictal activity in these two structures (EC/PC cut panel). **(B)** Two types of ictal discharge initiation can be recorded from the perirhinal cortex during bath application of 4-aminopyridine. Note that the ictal discharge shown in **(a)** is characterized by acceleration of the interictal events preceding its onset while that in **(b)** is characterized by a “slow” interictal event.

Experiments performed *in vitro* in extended brain slices comprising the hippocampus along with the entorhinal and perirhinal cortices have shown that interictal and ictal discharges are generated during bath application of the convulsant drug 4-aminopyridine or Mg^2+^ -free medium (de Guzman et al., 2004). These epileptiform patterns were only identified after severing the connections between these parahippocampal areas and the hippocampus; such a procedure abolished the propagation of CA3-driven fast interictal discharges that controlled the propensity of parahippocampal neuronal networks to generate “slow” interictal events along with prolonged ictal discharges (see for review, Avoli and de Curtis, 2011). As illustrated in Figure 3A, the epileptiform events recorded under control conditions from the entorhinal and perirhinal cortices occurred synchronously in these two areas, could initiate from any of them, and propagated to the neighboring structure with delays ranging from 8 to 66 ms. However, cutting the connections between entorhinal and perirhinal cortices generated independent epileptiform activity in both structures (Figure 3A, EC/PC cut); interestingly, these procedures shortened ictal discharge duration in the entorhinal but not in the perirhinal cortex. These experiments have also demonstrated that network synchronization underlying ictogenesis in the perirhinal cortex is N-Methyl-D-aspartate (NMDA) receptor-dependent (de Guzman et al., 2004).

We have recently reported that 4-aminopyridine-induced ictal discharges in the rat entorhinal cortex are preceded by an isolated “slow” interictal discharge or suddenly initiate from a pattern of frequent polyspike interictal discharges; only rarely ictal discharge onset was characterized by an acceleration of interictal event rates (Avoli et al., 2013). These findings contrast with what has been observed in the perirhinal cortex since retrospective analysis of the experiments published by de Guzman et al. (2004) indicates that in this area approximately half of the slices treated with 4-aminopyridine presented with ictal discharge onset characterized by acceleration of interictal events (Figure 3Ba) while in the remaining experiments ictal discharges are preceded by a “slow” interictal discharge (Figure 3Bb). These electrographic characteristics are reminiscent of the hypersynchronous onset and of the low-voltage, fast activity onset patterns, respectively, that have been reported to occur *in vivo* in both epileptic patients (Velasco et al., 2000; Ogren et al., 2009) and animal models (Bragin et al., 1999, 2005; Lévesque et al., 2012, 2013).

Overall, these *in vitro* data indicate that the perirhinal cortex may be more prone to generate ictal discharges as compared with the entorhinal cortex. In line with this view, *in vivo* studies have shown that kindling within the perirhinal cortex promotes seizure activity more rapidly than stimulation of the piriform cortex, amygdala or dorsal hippocampus (McIntyre et al., 1993, 1999; Sato et al., 1998). Moreover, lesioning the perirhinal cortex (Kelly and McIntyre, 1996; Fukumoto et al., 2002) or applying glutamatergic receptor antagonists (Tortorella et al., 1997) or adenosine A1 receptor agonists (Mirnajafi-Zadeh et al., 1999) to the perirhinal cortex attenuated and even prevented the appearance of seizure activity following amygdala kindling.

## Changes in excitability in pilocarpine-treated epileptic rats

By using *in vitro* electrophysiological recordings we have recently reported that brain slices obtained from pilocarpine-treated epileptic rats present with remarkable changes in synaptic excitability when compared to age-matched, non-epileptic controls (Benini et al., 2011). The pilocarpine model of temporal lobe epilepsy—which consists of an initial *status epilepticus* induced by i.p. injection of this cholinergic agonist that is followed 1–4 weeks later by a chronic condition of recurrent limbic seizures—is presumably the most commonly used model for studying this epileptic disorder (Curia et al., 2008). It provides the opportunity of controlling epilepsy severity and associated brain damage by pharmacologically regulating the duration of the initial *status epilepticus*. Moreover, in contrast to other chronic epilepsy models, spontaneous seizures recur frequently and consistently in virtually all pilocarpine-treated rats.

**Figure 4:**
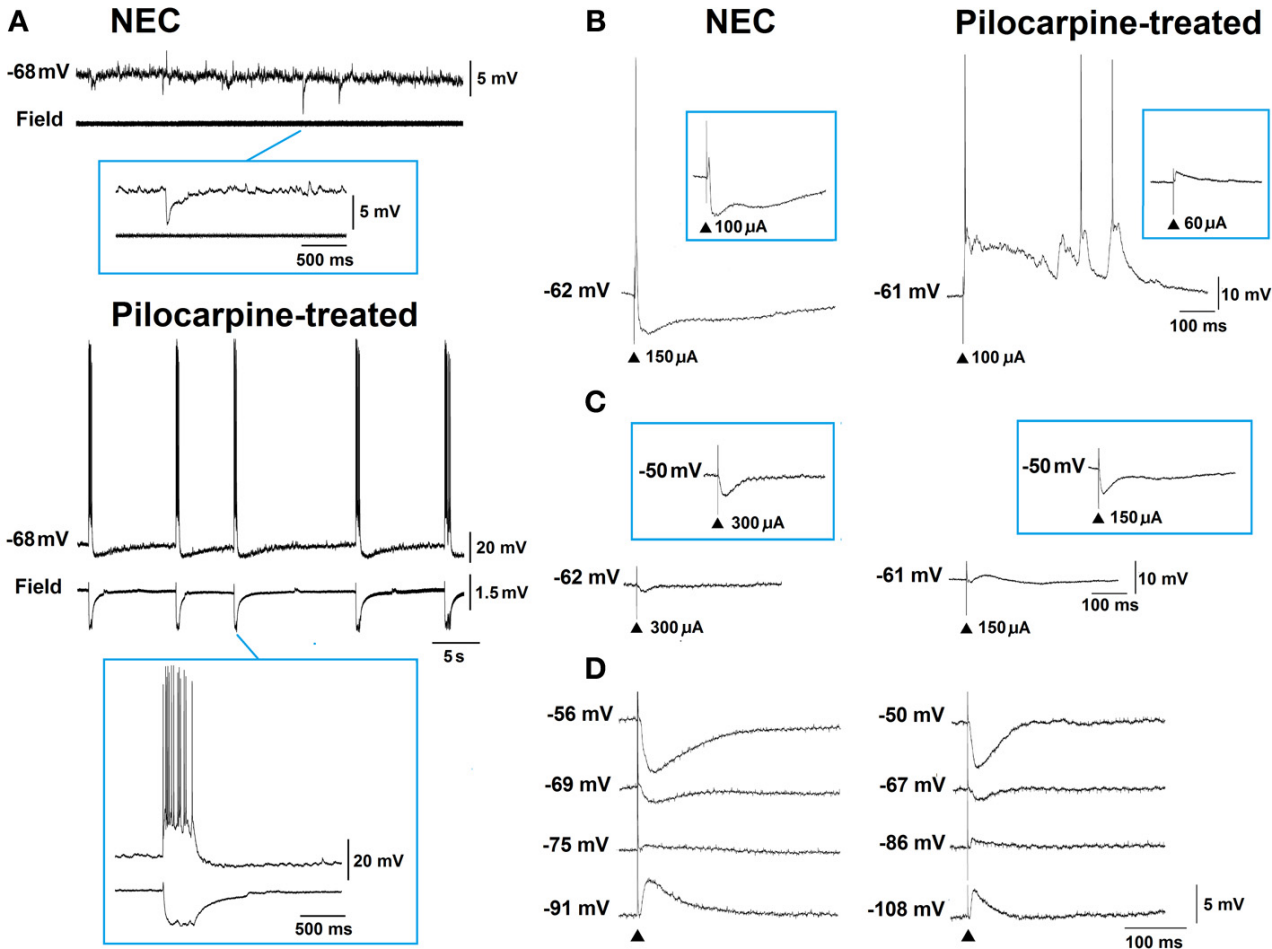
**(A)** Simultaneous field and intracellular recordings (−68 mV) in brain slices obtained from non-epileptic control (NEC) and pilocarpine-treated epileptic animals. Note in the NEC recording the presence of depolarizing and hyperpolarizing postsynaptic potentials (PSPs) while robust epileptiform activity occurs in the pilocarpine-treated experiment; expansion of the events is shown in the inserts. **(B)** and **(C)** Intracellular responses generated by perirhinal cortex neurons to local single-shock stimulation under control conditions and during blockade of glutamatergic transmission, respectively. Inserts illustrate the responses obtained by stimuli of lower strength **(B)** or during intracellular injection of steady depolarizing current **(C)**. **(D)** Intracellular responses induced by local single-shock stimulation in the presence of glutamatergic receptor antagonists. In this experiment the early γ-aminobutyric acid type A (GABA_A_) receptor-mediated component of the evoked inhibitory PSP was found to have a more depolarized reversal potential in the pilocarpine-treated neuron (−69.5 mV) as compared to the non-epileptic control cell (−74.7 mV).

Neurons recorded intracellularly from the deep layers of the perirhinal cortex of non-epileptic control and pilocarpine-treated animals had similar intrinsic and firing properties (Benini et al., 2011). Moreover, they generated spontaneous depolarizing and hyperpolarizing postsynaptic potentials with comparable duration and amplitude. However, spontaneous and stimulus-induced epileptiform discharges could be recorded with field potential and intracellular recordings in over one-fifth of pilocarpine-treated slices but never in control tissue (Figures 4A,B). These network events were reduced in duration by antagonizing NMDA receptors, and abolished by concomitant application of NMDA and non-NMDA glutamatergic receptor antagonists (Benini et al., 2011).

As illustrated in Figure 4C, electrical stimuli delivered during blockade of glutamatergic transmission induced IPSPs in perirhinal neurons recorded in both control and pilocarpine-treated brain slices. However, analysis of these stimulus-induced IPSPs revealed that the reversal potential of the early, GABA_A_ -receptor-mediated component was significantly more depolarized in pilocarpine-treated vs. control cells (Figure 4D) while no difference in peak conductance was identified (Benini et al., 2011). These differences are presumably caused by a decrease in the expression of the potassium-chloride cotransporter 2 that leads to a dysfunction in the balance of intracellular chloride. Indeed, we have found that immunoreactivity for the potassium-chloride cotransporter 2 is consistently lower in pilocarpine-treated epileptic rats, both in the perirhinal cortex (Benini et al., 2011) and in other parahippocampal regions (de Guzman et al., 2006).

## Interneurons are selectively damaged in the pilocarpine model of temporal lobe epilepsy

**Figure 5:**
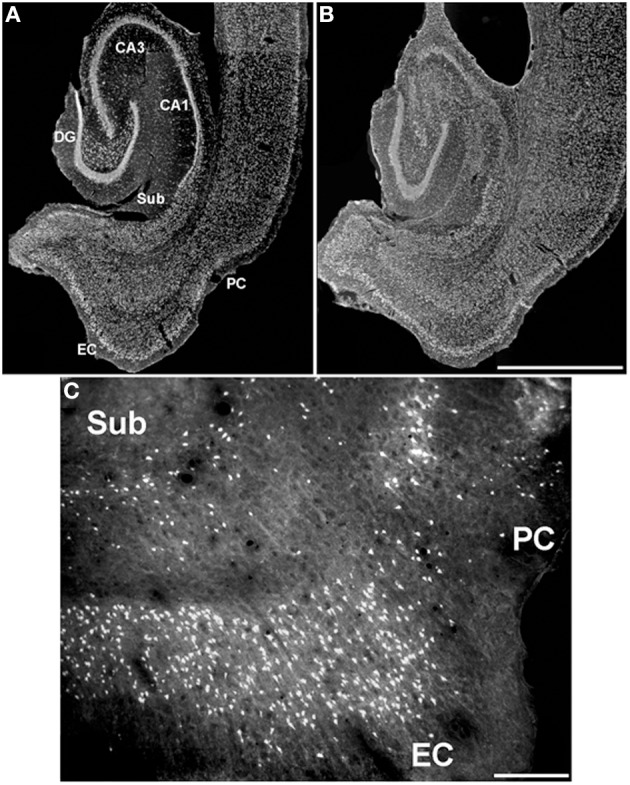
**Characterization of the neuronal damage occurring in the hippocampus and in parahippocampal structures in pilocarpine-treated rats**. Rats were treated and processed as described in Biagini et al. (2005). **(A** and **B)** dark field photomicrographs of Nissl-stained brain sections obtained from a non-epileptic control rat and a pilocarpine-treated rat, respectively. Note that remarkable damage is localized to the Cornu Ammonis (CA) subfield 1 (CA1), hilus of the dentate gyrus (DG), layer III of medial entorhinal cortex (EC), whereas the subiculum (Sub) and perirhinal cortex (PC) are apparently spared. **(C)** Section stained by Fluoro-Jade to identify dying cells shows the presence of necrotic neurons also in the perirhinal cortex, but to a lower extent than in the entorhinal cortex. Scale bars, 1,900 μm for **(A)** and **(B)**, 200 μm for **(C)**.

Substantial damage to perirhinal cortex has been reported in an animal model based on electrically induced *status epilepticus* (Bumanglag and Sloviter, 2008). However, injury to this limbic area has rarely been documented in rats treated with lithium-pilocarpine, in which neuronal cell counts were similar to control animals or non-significantly decreased by ~ 10% (André et al., 2000); in this study, neuronal damage in the perirhinal cortex became evident only when rats were exposed to electroshocks preceding the lithium-pilocarpine treatment (André et al., 2000). We were also unable to demonstrate any consistent damage to the perirhinal cortex in rats exposed to various durations of pilocarpine-induced *status epilepticus* (Benini et al., 2011; Gualtieri et al., 2012), a finding further confirmed by staining necrotic cells with Fluoro-Jade (Figure 5) (Biagini et al., 2005, 2008). These experimental findings are at odds with clinical data showing that the perirhinal cortex presents with consistent asymmetries when the region ipsilateral to the sclerotic hippocampus is compared with the contralateral (Bernasconi et al., 2000, 2003; Salmenperä et al., 2000; Jutila et al., 2001; O'Brien et al., 2003; Alessio et al., 2006; Guedj et al., 2010). These discrepancies may have several explanations. For instance, it should be considered that the time span between the precipitating injury and histopathological analyses is much shorter in experimental models than in clinical studies; hence, if cell damage in the perirhinal cortex requires more time than in the hippocampus or entorhinal cortex, most animal models would probably fail in detecting these changes.

Interestingly, a different scenario emerges when specific cell types are analyzed in the perirhinal cortex of pilocarpine-treated epileptic rats. We have reported that ~ 20 weeks after pilocarpine treatment parvalbumin (PV), neuropeptide Y (NPY) and cholecystokinin (CCK) immunopositive interneurons are significantly decreased in adult rats (Benini et al., 2011). The loss of interneurons in absence of a corresponding reduction in principal cells may lead to a profound alteration in the functional characteristics of this brain structure (de Guzman et al., 2006, 2008; Benini et al., 2011). Whereas in control tissue cells immunopositive for PV were clearly prevalent (68% of all stained interneurons) when compared with other putative interneurons (25 and 7% of all stained interneurons were NPY or CCK immunopositive, respectively), in pilocarpine-treated rats PV immunopositive cells decreased to 56% of all stained interneurons, whereas NPY and CCK immunopositive cells increased to 32 and 12%, respectively (Benini et al., 2011). Further evaluation of these interneuronal subpopulations also revealed that the superficial perirhinal cortical layers of pilocarpine-treated rats contain more interneurons than analogous layers in control non-epileptic rats (53% vs. ~ 48% of all stained interneurons) (Benini et al., 2011). These data are in line with those obtained from other limbic regions of epileptic rats, such as the hippocampal CA1 subfield (André et al., 2001) and the dentate hilus (Gorter et al., 2001). Interestingly, PV immunopositive cells are also decreased in the neocortex (DeFelipe et al., 1993) and hippocampus (Arellano et al., 2004) of epileptic patients presenting with intractable seizures. Inhibitory networks within the perirhinal and entorhinal cortices confer these structures with the ability to actively gate signal transmission between the neocortex and the hippocampus (Biella et al., 2002; de Curtis and Paré, 2004; Pelletier et al., 2004). These functional characteristics may be relevant for controlling the spread of epileptiform activity within the limbic system and for understanding the role played by decreased inhibition in the perirhinal cortex of epileptic patients and animals.

**Figure 6:**
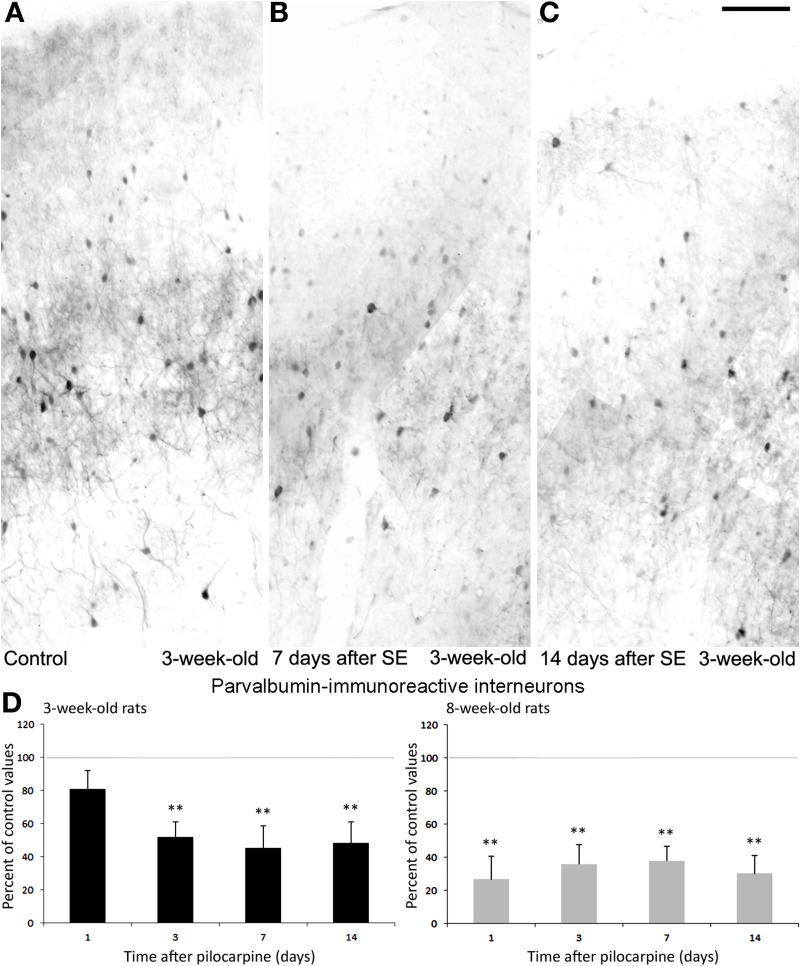
**Parvalbumin (PV)-immunopositive interneurons in the rat perirhinal cortex**. Photomicrographs showing interneurons stained with an antibody against PV in the perirhinal cortex of 3-week-old rats **(A–C)**. Specifically, PV immunostaining is shown in a control, non-epileptic rat **(A)**, and in pilocarpine-treated rats 7 **(B)** and 14 days **(C)** after pilocarpine treatment. **(D)** Normalized (respect to control) quantification of PV immunostained neurons in 3 and 8-week-old rats following pilocarpine treatment. Note in the 3-week-old animals (*n* = 3–6 for each time interval) that PV immunostained neurons decrease significantly 3 days as well as 7 and 14 days later. Note that similar findings were observed in 8-week-old rats (*n* = 4–5 for each time interval). ^**^p < 0.01, analysis of variance followed by Tukey's test for multiple comparisons. Scale bar: 100 μm. Animal treatment is described in Biagini et al. (2008). Details of the immunostaining procedure and cell counts are in de Guzman et al. (2006, 2008); Bortel et al. (2010) and Benini et al. (2011).

Recently, we have also studied interneuron damage in rats exposed to pilocarpine-induced *status epilepticus* at 3 weeks of age; these animals are more resistant to damage and have a tendency to develop chronic seizures of lower severity, compared with 8-week-old rats (Biagini et al., 2008). Results obtained from adult rats confirmed our previous findings regarding a decrease in PV-positive cells (Figure 6; cf., Benini et al., 2011). In addition, although rats exposed to pilocarpine-induced *status epilepticus* at 3 weeks of age were less prone to develop neuronal damage (Biagini et al., 2008), we found approximately a 50% decrease in PV interneurons 3 days after the pilocarpine administration; such reduction was maintained in the following time intervals of 7 and 14 days (Figures 6A–D), suggesting that this interneuron subtype is very sensitive to damage and that this occurs independently of brain maturation.

**Figure 7:**
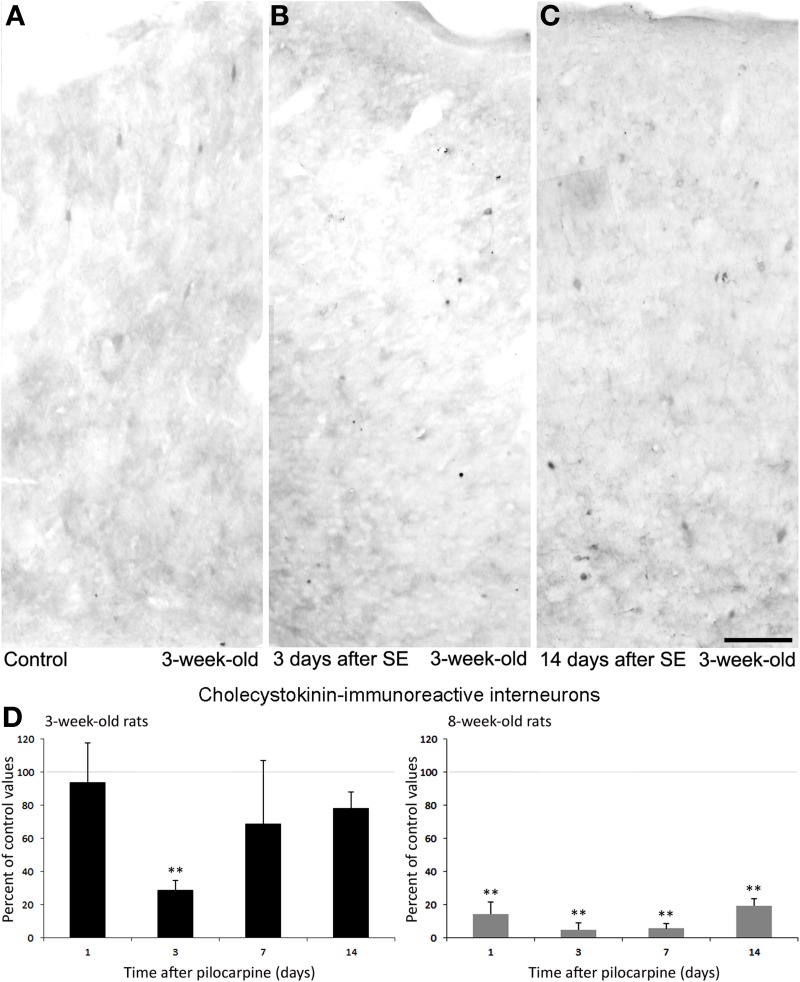
**Cholecystokinin (CCK)-immunopositive interneurons in the rat perirhinal cortex**. Photomicrographs showing interneurons stained with an antibody against CCK in the perirhinal cortex of 3-week-old rats **(A–C)**. Specifically, CCK immunostaining in a control, non-epileptic rat, **(A)** and in pilocarpine-treated rats 3 **(B)** and 14 days **(C)** after pilocarpine treatment are respectively shown. **(D)** Normalized (respect to control) quantification of CCK immunostained neurons in 3 and 8-week-old rats following pilocarpine treatment. Note in the 3-week-old animals (*n* = 3–6 for each time interval) that CCK immunostained neurons decrease significantly 3 days after pilocarpine but recovered 7 and 14 days later. Note also that a consistent decrease in CCK-positive neurons was observed in 8-week-old rats (*n* = 4–5 for each time interval). ^**^p < 0.01, analysis of variance followed by Tukey's test for multiple comparisons. Scale bar: 100 μm. Animal treatment is described in Biagini et al. (2008). Details of the immunostaining procedure and cell counts are as in Benini et al. (2011) and Gualtieri et al. (2013).

At variance with PV-immunopositive cells, CCK interneurons in the perirhinal cortex showed transitory changes in 3-week-old rats. This phenomenon, which may reflect functional adaptation to *status epilepticus* rather than cell damage, was limited to young rats whereas adult rats presented merely with loss of interneurons. As shown in Figures 7A–D, interneurons stained by an antibody against CCK (Benini et al., 2011; Gualtieri et al., 2013) were significantly (*p* < 0.01) reduced in 3-week-old rats at day 3 after pilocarpine treatment, but counts of these interneurons were comparable to control values at days 7 and 14 after *status epilepticus*. This finding could be related to a transient impairment in CCK synthesis or to an increased release. In contrast with the time course observed in the young group of animals, 8-week-old rats presented a strong reduction in CCK immunopositive cells to ~ 20% of control values, which was found at every considered time point (Figure 7D). Interestingly, these results highlight a different age-related sensitivity of CCK interneurons to pilocarpine-induced *status epilepticus* by confirming an enhanced resilience to damage in young animals. It remains to be established, however, whether this difference could be related to the lower propensity of young rats to develop recurrent generalized seizures when exposed to *status epilepticus*, in contrast to what observed in adult animals (Biagini et al., 2008).

**Figure 8:**
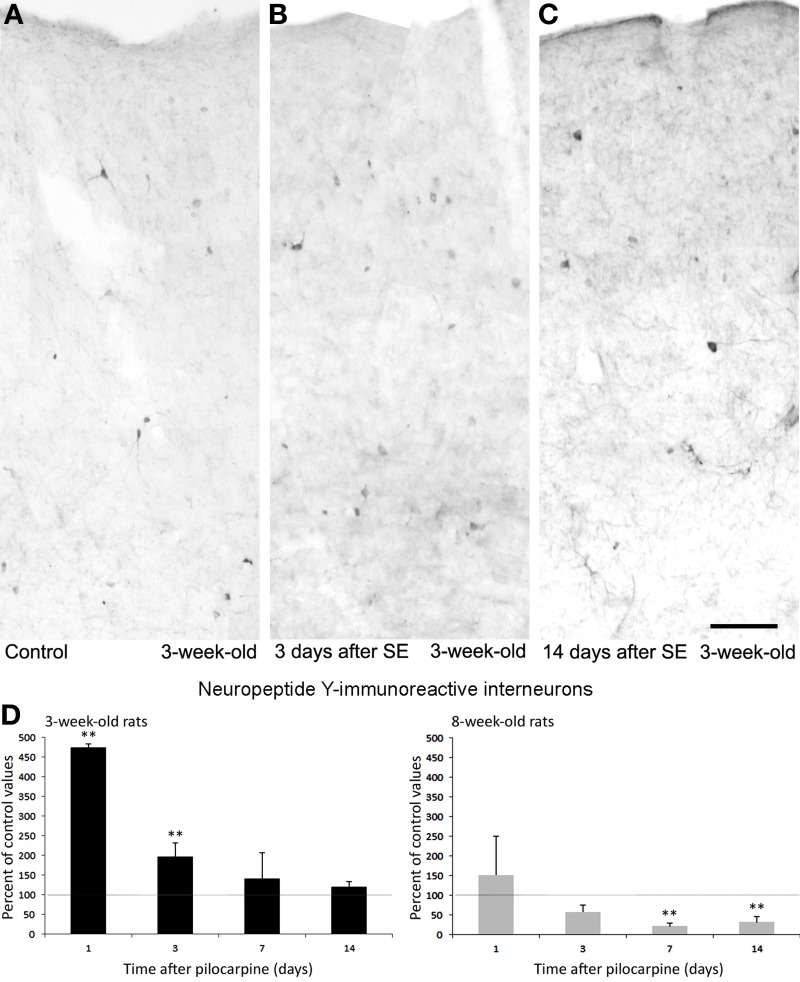
**Neuropeptide Y (NPY)-immunopositive interneurons in the rat perirhinal cortex**. Photomicrographs showing interneurons stained with an antibody against NPY in the perirhinal cortex of 3-week-old rats **(A–C)**. Specifically, NPY immunostaining is shown in a control, non-epileptic rat **(A)** and in pilocarpine-treated rats 3 **(B)** and 14 days **(C)** after pilocarpine treatment. **(D)** Normalized (respect to control) quantification of NPY immunostained neurons in 3 and 8-week-old rats following pilocarpine treatment. Note in the 3-week-old animals (*n* = 3–6 for each time interval) that NPY immunostained neurons increased significantly 1 and 3 days after pilocarpine, but recovered 7 and 14 days later. Note that a consistent decrease in NPY interneurons was instead observed in 8-week-old rats (*n* = 4–5 for each time interval). ^**^p < 0.01, analysis of variance followed by Tukey's test for multiple comparisons. Scale bar: 100 μm. Animal treatment is described in Biagini et al. (2008). Details of the immunostaining procedure and cell counts are as in Bortel et al. (2010); Benini et al. (2011) and Gualtieri et al. (2013).

As illustrated in Figures 8A–D, interneurons expressing NPY transiently increased 1 day after *status epilepticus* induction both in young and adult rats, but in the latter group the changes were not large enough to be statistically significant. In young rats, counts of NPY interneurons decreased to basal values a week later and were maintained at normal levels also 14 days after pilocarpine treatment (Figure 8C). Thus, the transient, functional changes occurring in the counts of NPY interneurons may be related to an increased synthesis of this neuropeptide, or to a block of its release. In contrast to these findings, adult rats presented a steady decrease of NPY interneurons to less than 50% of basal values (Figure 8D), thus confirming our previous observations (Benini et al., 2011). These age-dependent discrepancies further support the hypothesis that acute functional changes occur, as in the case of CCK interneurons, also for other interneuronal subpopulations. In addition, this evidence underscores age-related differences; specifically, interneurons are more preserved after exposure to *status epilepticus* in young rats than in adult animals.

Changes in the ratio of interneurons and principal cells may play a role in epileptogenesis, as suggested by Tuunanen et al. (1997). However, we recently obtained evidence of a similar selective alteration in subpopulations of interneurons with limited changes in neuronal excitability in the insular cortex (Bortel et al., 2010). The differences found in the various subpopulations of interneurons between the two groups of young, 3-week-old rats and adult animals may also confirm the involvement of interneuron loss in epileptogenesis (Tuunanen et al., 1997; Gorter et al., 2001). Young animals with preserved CCK and NPY interneurons (Figures 7, 8) were less prone to develop generalized convulsive seizures than adult rats (Biagini et al., 2008), in which the same interneurons were markedly decreased. Therefore, it is likely that these interneuronal subclasses contribute to maintain the perirhinal cortex under a rather physiological condition. In contrast, since similar levels of PV interneuron loss were found in young and adult rats exposed to pilocarpine-induced *status epilepticus*, we are inclined to hypothesize a less critical role of these interneurons in modulating the propensity of perirhinal cortex neuronal networks to generate chronic seizure following the initial *status epilepticus* induced by pilocarpine.

## Conclusive remarks

Perhaps, one of the most essential developments in temporal lobe epilepsy research in the last few years has been the recognition that the pathophysiological substrates underlying this neurological disorder extend beyond the hippocampus to involve not only extrahippocampal but extratemporal structures as well (De Carli et al., 1998; Lee et al., 1998; Sandok et al., 2000; Dreifuss et al., 2001; Moran et al., 2001; Natsume et al., 2003; Seidenberg et al., 2005). Advances in neuroimaging techniques have revealed that volumetric reductions of the amygdala, entorhinal and perirhinal cortices do occur in a subset of patients affected by temporal lobe epilepsy in spite of normal hippocampal volumes (Cendes et al., 1993; Bernasconi et al., 1999, 2001, 2003; Salmenperä et al., 2000; Jutila et al., 2001). Histopathological examination of human epileptic tissue have also corroborated these findings by demonstrating the presence of selective neuronal loss and synaptic reorganization within these structures even in the absence of hippocampal sclerosis (Du et al., 1993; Hudson et al., 1993; Miller et al., 1994; Wolf et al., 1997; Mikkonen et al., 1998; Yilmazer-Hanke et al., 2000; Aliashkevich et al., 2003). The data reported in this review provide evidence that supports the view that the perirhinal cortex may be implicated in the processes of epileptogenesis and ictogenesis. Our data showing a clear role of the perirhinal cortex in the onset of seizure-like discharges *in vitro*, indicate that this structure has the capacity to generate ictal events and to reproduce in several cases the so-called hypersynchronous seizure onset pattern. In addition, the decrease of specific interneuronal subclasses in the perirhinal cortex of pilocarpine-treated rats suggests that the impairment of perirhinal functions may have been overlooked by simply considering the gross anatomical changes that occur in this region in temporal lobe epilepsy patients. In this respect, it is enticing to propose that the main network function of the perirhinal cortex may consist of a strong inhibitory control exerted on the entorhinal cortex, presumably mediated by GABAergic neurons located in layers III and IV of areas 35 and 36 (Apergis-Schoute et al., 2007). The prominent decrease of interneurons stained by PV, CCK and NPY antibodies observed in the perirhinal cortex of epileptic rats, including its superficial layers (Benini et al., 2011), could have a very important role in determining the hyperexcitability consistently observed in the entorhinal cortex of pilocarpine-treated rodents (D'Antuono et al., 2002; de Guzman et al., 2008; Panuccio et al., 2010). Further exploration of these brain regions is necessary for identifying their specific roles in the initiation and spread of seizures in temporal lobe epilepsy. Finally, it is worthwhile to mention that a detailed assessment of extrahippocampal structures in temporal lobe epilepsy might help to increase our understanding of the mechanisms underlying the pathophysiology of this neurological disorder as well as the functional changes that occur within these limbic areas during epileptogenesis.

### Conflict of interest statement

The authors declare that the research was conducted in the absence of any commercial or financial relationships that could be construed as a potential conflict of interest.
